# Tobacco control in the context of the COVID-19 pandemic in Uganda: a policy implementation review

**DOI:** 10.29392/001c.17607

**Published:** 2020-12-08

**Authors:** Steven Ndugwa Kabwama, Kellen Namusisi Nyamurungi, Fiona Davidson, Fiona Dobbie, Linda Bauld

**Affiliations:** 1 Makerere University School of Public Health, College of Health Sciences, Kampala Uganda; 2 Makrere University School of Public Health; 3 Usher Institute, University of Edinburgh

**Keywords:** covid-19, tobacco policy, tobacco use

## Abstract

**Background:**

The COVID-19 pandemic has caused more than 900,000 deaths globally. The risk of mortality is higher for people with pre-existing conditions such as cancers, respiratory and cardiovascular diseases and diabetes for which tobacco use is a known risk factor. We conducted a study to explore how efforts to address the COVID-19 pandemic in Uganda have been integrated with tobacco control policies to generate evidence to inform policy decisions about the public health response in general and tobacco control interventions in particular.

**Methods:**

We conducted a desk based review of ‘grey’ literature data sources (i.e. data that were not included in peer reviewed journals) with information about tobacco and COVID-19 in Uganda. Data were also obtained from stakeholders involved tobacco control via an online survey and telephone interviews.

**Findings:**

A total of 136 data sources were identified, of which 107 were eligible for data extraction. The online stakeholder consultation involved invitations to 61 participants of whom 33 (54%) took part via the online survey while 5 (8.2%) opted for telephone interviews. In the context of the COVID-19 prevention interventions, social media can be a powerful platform for communicating anti-tobacco messages such as the vulnerability of tobacco users to COVID-19 and the exacerbated disease severity among COVID-19 patients with history of tobacco use. Two thirds (n=20, 65%) of survey respondents expected a tobacco tax increase to address health, economic and wider policy impacts of the COVID-19 crisis.

**Conclusions:**

Advocacy should be conducted for taxation of tobacco products to reduce consumption and generate revenue to support public health investments. Public health institutions involved in the COVID-19 response should reject donations from the tobacco industry and its allies as is stipulated in the Framework Convention on Tobacco Control and the Uganda Tobacco Control Act 2015. The COVID-19 pandemic also offers an opportunity to promote tobacco cessation and strengthening tobacco control policy implementation by recognizing the role of tobacco use in exacerbating COVID-19 health outcomes.

By 31 October 2020, the COVID-19 pandemic had affected over 45 million people and caused 1 million deaths globally.^1^ The risk of severe morbidity and death has been shown to be higher among males, older people and those with underlying non-communicable disease conditions such as respiratory and cardiovascular diseases and cancer.^2^ Tobacco use is a major risk factor for the development of many of the underlying disease conditions such as cancers, respiratory and cardiovascular diseases as well as diabetes.^3^ In addition, exposure to tobacco smoke and nicotine affects lung development and impairs the body’s immune response to viral infection.^4^ The causative agent of COVID-19 is the Severe Acute Respiratory Coronavirus 2 (SARS-Cov-2) which enters the body through the nose, mouth and upper respiratory tract. On the other hand, exposure to tobacco smoke leads to inflammation of the mucosal tissues in the upper respiratory tract.^5^ Tobacco use has thus been identified as a risk factor for critical/ mortal disease progression among patients with COVID-19.^2^ The association between tobacco use and COVID-19 is such that there is an increased risk of COVID-19 infection due to a suppressed immune system as well as the exacerbation of symptoms and disease severity among COVID-19 patients with non-communicable diseases and a history of tobacco use. In Uganda, the 2013 Global Adult Tobacco Survey (GATS) reported that more than 2.5 million people were current tobacco users while over 4.5 million people had been exposed to tobacco smoke.^6^ By 31 October 2020, Uganda had recorded >10,00 cumulative confirmed cases and >100 deaths^1^ due to COVID-19. It is thus critical for the COVID-19 public health response to be cognizant of the influence of tobacco use and tobacco smoke exposure. We conducted a study to explore how efforts to address the COVID-19 pandemic in Uganda have been integrated with tobacco control policies and interventions. Findings from this study provide evidence to inform policy decisions about the public health response in general, and tobacco control interventions in particular, in the context of infectious diseases like COVID-19. Recommendations from the study are relevant for Uganda in particular, other countries in Sub-Saharan Africa such as Tanzania^7–9^ and other low and middle income countries with similar tobacco control frameworks and tobacco use profiles.

## Methods

This was a mixed method study with data collected in three ways. First, we conducted a desk-based mapping of ‘grey’ literature data sources (i.e. data that were not included in peer reviewed journals, such as government reports, online print press and Twitter) on COVID-19 and tobacco. Delivery of the desk-based mapping was guided by a protocol which is included as a supplementary file. In summary our data search was web-based via a Google search using the “All” and “News” tab with the search terms ‘COVID-19’, ‘smoking’, ‘smokeless tobacco’, and ‘tobacco industry’. Websites of the Uganda Government, Uganda Ministry of Health, WHO Framework Convention on Tobacco Control (FCTC), and WHO Country Office for Uganda were also reviewed for published information related to tobacco and COVID-19. Websites of non-governmental organizations involved in tobacco control activities in Uganda such as the Center for Tobacco Control in Africa, AMREF Health Africa, African Tobacco Control Alliance, Tobacco Atlas, Tobacco Control Uganda and the Uganda Health Communication Alliance were also reviewed for information relevant to the objectives of the study. The social media accounts of the Uganda Ministry of Health, Uganda Government, WHO Uganda, Tobacco Control Uganda and the Uganda Health Communication Alliance were also reviewed for information related to tobacco use and COVID-19. The desk-based mapping search was conducted thrice. Initially on 15 May 2020, we searched for data sources published between the date the WHO declared COVID-19 a public health emergency of international concern (30 January 2020) and 1 June 2020. The second search was conducted on 22 June 2020 and the final search on 1 July 2020. A total of 136 online and offline data sources were identified of which 107 (78.7%) were eligible for data extraction. As per the protocol, data sources were screened to assess whether they met the eligibility criteria, with data extraction recorded using Microsoft excel. The majority of data sources from came from the Twitter accounts of Tobacco Control Uganda and the Uganda Health Communication Alliance and mostly took the form of advice or information sharing. For example, advising tobacco users of their potentially higher risk from COVID-19, as well as the impact of COVID-19 on tobacco control and the role of the tobacco industry in response to COVID-19.

Next, we consulted with key stakeholders involved in non-communicable diseases control in general and tobacco control in particular. For ease of completion there were offered given the option to take part via an online survey or telephone interview. Each mode of data collection collected the same level of detail, but as would be expected telephone interviews gave more flexibility to expand on answers and, in some cases, provided greater depth of detail. Stakeholders who had attended previous non-communicable diseases control engagements such as development of national policies and strategic action plans, were invited to participate in the survey. Stakeholder consultation covered issues to do with the relationship between COVID-19 and tobacco use, impact of the COVID-19 pandemic on the policy response to tobacco control and implications of the COVID-19 pandemic on development of tobacco control policy. The qualitative data and open ended response from the survey were analysed using a systematic step-by-step review of the data and identification of themes related to the study topic. Descriptive analysis was conducted for survey findings and are presented as proportions. The online stakeholder consultation involved invitations to 61 participants of whom 33 (54%) took part via the online survey while 5 (8.2%) opted for telephone interviews. The institutional affiliations of the respondents were civil society (n=14, 36.8%), government (n=12, 31.6%), research/ academic institutions (n=7, 18.4%) and others (n=5, 13.2%).

## Key Findings

As noted above, the first stage of analysis looked at each strand of the study separately. We then triangulated findings to generate the key findings from the study. These are now presented, with recommendations for future policy also discussed.

### Covid-19 Advice to Tobacco Users and Impact on Tobacco Control Policy

Six out of ten survey respondents (n=20, 61%) agreed that the relationship between COVID-19 and tobacco use had been discussed in policy contexts or social media particularly Twitter, Facebook, Instagram which suggests an awareness of COVID-19 and tobacco use among social media users. Results from the mapping also highlighted the importance of social media for conveying information about COVID-19 and tobacco with the main source of data being the Twitter accounts of Tobacco Control Uganda (TC Uganda) and the Uganda Health Communication Alliance (UHCA). The use of social media to disseminate public health messages has been shown to increase knowledge and attitudes^10^ and may offer a unique opportunity for the promotion of anti-tobacco messages.^11^ This could be especially relevant in the context of the COVID-19 pandemic where physical distancing is integral to the control of transmission. However, there was also the view that this issue had received limited attention:

“Generally, in my own observation, there is very limited focus/discussion on tobacco use and COVID 19.” (Interviewee)

Public information on COVID-19 and tobacco largely focused on the vulnerability of tobacco users to COVID-19 and their potentially higher risk of contracting COVID-19. The mapping identified multiple Tweets and re-tweets from TC Uganda and the UHCA advising the public that the hand to mouth contact involved in tobacco use could aid the spread of COVID-19. The UHCA also tweeted that both active and passive tobacco users are at greater risk of severe illness from COVID-19.

### Impact Of Covid-19 On Tobacco Taxation Policy

Respondents expressed a view that revenue to support the health sector and tobacco control could be generated by reviewing the current tobacco taxation policy and were generally in favour of a tax increase to replace the revenue lost to COVID-19. For example, nearly two thirds (n=20, 65%) of survey respondents expected a tobacco tax increase to address health and wider policy impacts of the COVID-19 crisis.

“The country is looking for ways to get more revenue and tobacco taxation has been identified as a potential area. Once taxes go high, the prices [for tobacco products] will also increase and thus reduced use among the poor and the young”

The Ministry of Health could achieve this by working with CSOs to advocate for the taxation of tobacco products to reduce consumption and generate revenue to support investments in public health. Although the COVID-19 interventions to interrupt transmission are expected to gravely affect global and national economies,^12^ tobacco taxation as a public health policy has the potential to generate revenue and reduce overall tobacco use by preventing and delaying initiation among young people and promoting cessation and lowering consumption among those who use.^13^

### Tobacco Industry and the National Response to the Covid-19 Pandemic

Four out of 10 survey respondents (n=13, 41%) and all stakeholders interviewed by telephone (n=5, 100%) thought that the tobacco industry had engaged in new corporate social responsibility activities, marketing or had sought to influence the policy response to COVID-19. Respondents reported that the COVID-19 pandemic had given the tobacco industry an opportunity to participate at national and district levels by donating US $65,000 to the National COVID-19 task force. Respondents also noted that the industry had made claims that nicotine had protective factors against COVID-19. The mapping identified several tweets from the Uganda Health Communication Alliance and Tobacco Control Uganda refuting the claim.

"The Tobacco industry donated funds to COVID-19 response fund for Uganda. It even got a presidential mention as recognition for the ‘act of good will’.

Elsewhere, an analysis of social media data from the United States between January and May 2020 revealed that the tobacco industry had made deliberate efforts to promote messages such as the tobacco industry supporting the development of a COVID-19 vaccine and disputing the association between tobacco use and COVID-19.^14^ Ministry of Health and civil society partners should provide the public with information dissociating the tobacco industry with the COVID-19 response and remind leadership and management of public institutions to reject donations from the tobacco industry as guided by the Framework Convention on Tobacco Control and stipulated in the 2015 Uganda Tobacco Control Act.^15^

### Advice About Tobacco Use Cessation

The main type of COVID-19 advice was to stop smoking or using tobacco products, which was mostly provided by the Ministry of Health and civil society officials. This provides an opportunity to promote tobacco use cessation in Uganda. However, survey findings suggest that respondents did not see a change in the level of interest in tobacco use cessation among users, with 26 (82%) reporting no change or saying that they did not know. Tobacco control efforts should leverage the fight against COVID-19 and push for reductions in tobacco use and promote tobacco use cessation by emphasizing the effects of the two conditions on the lungs and the additional mortality risk among COVID-19 patients who are current or former tobacco users compared to those that have never used tobacco. Preliminary research has demonstrated that even 4 weeks of tobacco use cessation could reduce the risk of adverse outcomes related to COVID-19 such as intubation.^16^ Tobacco control messages should thus be disseminated along with social distancing and sanitation and hygiene messages for the prevention of COVID-19.

### Opportunities for Tobacco Control Policy Implementation

Respondents were hopeful that the COVID-19 pandemic offered an opportunity to strengthen tobacco control policy implementation:

“I think it’s the best time to strengthen and implement the tobacco control policies. The effects of COVID-19 are immense on tobacco users and this should be put forward”

However, there was also the view that tobacco control had been neglected due to the pandemic. Respondents were aware that COVID-19 would have negative implications for the tobacco control policy agenda in Uganda, such as reduced funding, heightened tobacco industry influence and possible amendments to the Tobacco Control Act.

### Additional Information to Support Tobacco Control Policy

Survey and interview respondents highlighted that the following information would be helpful in the formulation of tobacco control policy in the context of COVID-19:

1. Understanding how the COVID-19 pandemic has affected tobacco consumption.
2. A better understanding of the relationship between tobacco use and COVID-19 (i.e. medical effects, disease progression, recovery time, mortality and transmission)
3. Assessment of tobacco industry interference during COVID-19 pandemic
4. An evaluation of the economic cost of COVID-19 on tobacco control programmes

## Conclusions

Tobacco use results in pre-mature mortality and morbidity globally to an extent that far exceeds that of COVID-19. Global deaths from direct tobacco use account for 7 million people per year.^17^ While tobacco use in Uganda is lower than in many other countries, it is imperative that national governments attend to their responsibility to prevent premature deaths from tobacco, and the associated morbidity by developing and implementing policies that reduce and prevent tobacco use. This is particularly important at the current time when tobacco use can, from existing evidence, affect COVID-19 disease severity. Countries need to integrate interventions and messages to avert communicable and non-communicable disease risks at the current time for the overall health and wellbeing of their populations.
